# Artificial intelligence in perioperative pressure injury management: a scoping review of evidence, gaps, and implications for population health and digital public health policy

**DOI:** 10.3389/fpubh.2026.1952864

**Published:** 2026-10-01

**Authors:** Qin Zeng, Jiawei Li, Jiacheng Hu, Cheng Yang

**Affiliations:** 1 Department of Pediatric Gastroenterology Nursing, West China Second University Hospital, Sichuan University, Chengdu, China; 2 Key Laboratory of Birth Defects and Related Diseases of Women and Children, Sichuan University, Ministry of Education, Chengdu, China; 3 Department of Pediatric Outpatient Nursing, West China Second University Hospital, Sichuan University, Chengdu, China; 4 Department of Nursing, West China School of Nursing, Sichuan University, Chengdu, Sichuan Province, China; 5 Department of Pediatric Intensive Care Unit Nursing, West China Second University Hospital, Sichuan University, Chengdu, China; 6 Department of Pediatric Intensive Care Unit Nursing, WCSUH-Tianfu·Sichuan Provincial Children’s Hospital, Meishan, China

**Keywords:** artificial intelligence, digital public health, health policy, patient safety, perioperative period, pressure injury, risk prediction, scoping review

## Abstract

**Background:**

Perioperative pressure injury (POPI) is a significant global public health challenge, contributing to substantial iatrogenic morbidity and heavy economic burden worldwide. Traditional prevention is limited by insufficient assessment tools, the “black box” nature of intraoperative monitoring, and delayed intervention. Artificial intelligence (AI) offers a novel approach by integrating multidimensional data for real-time risk prediction; however, no scoping review has systematically mapped AI applications across the full perioperative continuum from a population health perspective.

**Methods:**

Systematic searches were conducted in PubMed, Embase, CINAHL, Cochrane Library, IEEE Xplore, ACM Digital Library, Web of Science, and Scopus from inception to December 11, 2025. Studies applying machine learning, conventional regression-based benchmarking tools, or intelligent sensor-based monitoring for POPI prediction, monitoring, or prevention during the perioperative period were included. Two reviewers independently performed screening and data extraction, followed by descriptive synthesis and evidence mapping.

**Results:**

Six studies (2018–2025), all from East Asia, were included. This geographic concentration highlights an unassessed generalizability and algorithmic fairness risk, rather than an established inequity finding. Evidence concentrated on post-hoc risk stratification (five prediction studies), predominantly using machine learning models (e.g., Random Forest, XGBoost) with AUCs of the best-performing models ranging from 0.806 to 0.836; one study reported a prediction accuracy of 0.9733, which is not directly comparable to AUC. Models identified specialty-specific risk factors (e.g., cardiopulmonary bypass time, forced positioning). Only one proof-of-concept study addressed intraoperative real-time monitoring, visualizing pressure increases after 2 hours of surgery. Evidence for postoperative early warning systems is absent.

**Conclusion:**

Current studies demonstrate the feasibility of specialty-specific post-hoc risk stratification using intraoperative covariates, though truly preoperative models remain absent. Intraoperative monitoring shows early promise, but postoperative early warning is a critical gap, and evidence lacks global diversity. Future research should develop closed-loop intelligent systems integrating preoperative prediction, intraoperative multimodal monitoring, and postoperative warning. Nursing protocols should consider incorporating AI-identified risk factors as hypothesis-generating inputs pending prospective validation, while policymakers should establish frameworks for continuous validation and cross-population calibration to ensure equitable impact.

## 1 Introduction

Perioperative pressure injuries (POPIs) refer to localized ischemic and hypoxic tissue damage resulting from prolonged immobility or fixed positioning during the preoperative, intraoperative, and early postoperative periods (1). As a leading cause of iatrogenic harm worldwide, POPIs represent a major public health priority. The overall risk among surgical patients remains persistently high, with incidence rates rising significantly in prolonged, high-risk procedures such as cardiac and spinal surgeries—reaching up to 33.3% in some cohorts (2, 3). These injuries prolong hospital stays, increase readmission rates, and impose a substantial financial burden on healthcare systems and society (4, 5). Effective prevention is therefore not merely a clinical quality indicator but a critical component of population health management and health system sustainability.

Current prevention strategies center on preoperative risk assessment using tools such as the Braden and Munro Scales, alongside standardized nursing interventions including pressure-relieving positioning pads. Yet these approaches have notable limitations within the operating room. Generic risk assessment tools lack sensitivity to dynamic intraoperative factors—such as anesthesia duration, blood loss, and hemodynamic instability—that are known to influence POPI development (1). Moreover, sterile draping renders the patient’s skin invisible to the clinical team, creating a monitoring “black box” that precludes direct observation of early tissue changes (6). Interventions guided by static preoperative assessments cannot adapt to sudden physiological shifts during surgery, often resulting in delayed action (7). Collectively, these limitations underscore an urgent need for methods that synthesize multidimensional data and provide real-time risk awareness and early warning.

Artificial intelligence offers a promising avenue for addressing these challenges. Machine learning algorithms can extract non-linear risk patterns from high-dimensional electronic health record data, enabling more accurate risk stratification (7). Internet of Things sensors and computer vision technologies can quantify interface pressure in real time, monitor the skin microenvironment, and detect early erythema—capabilities that could transform POPI prevention from a reactive to a dynamic process (8).

Although AI is increasingly applied to pressure injury prevention, existing reviews have not adequately addressed the perioperative context. Some focus on patients in ICUs or general wards, thereby missing the distinctive physiological and procedural features of the surgical setting (9, 10). Others examine perioperative pressure injuries but emphasize manual prevention techniques with limited attention to intelligent technologies (11, 12). To date, no review has systematically mapped AI technologies across the full perioperative workflow—from preoperative prediction to intraoperative monitoring to postoperative surveillance (13, 14).

Unlike previous reviews focused on specific clinical settings or individual technologies (9–4), this scoping review applies a perioperative continuum framework and a population health and policy lens to AI-enabled POPI management. Our objectives are to: ① identify the types of AI technologies used in this field; ② describe their roles across the perioperative care pathway (preoperative, intraoperative, and postoperative); ③ summarize their reported performance and implementation outcomes; and ④ clarify existing research gaps to guide future development and clinical translation, with the goal of informing population-level prevention strategies and health policy. By mapping the evidence landscape and identifying gaps in real-time surveillance and early warning, this review directly responds to digital public health priorities for leveraging connected data systems to enhance patient safety and equitable risk stratification.

## 2 Methods

This scoping review was conducted in accordance with the methodological framework established by the Joanna Briggs Institute (JBI) for scoping reviews. Reporting followed the Preferred Reporting Items for Systematic Reviews and Meta-Analyses extension for Scoping Reviews (PRISMA-ScR) guidelines. A completed PRISMA-ScR checklist is provided in Supplementary material S2.

### 2.1 Study design and reporting guidelines

The PCC (population, concept, context) model guided the development of the following research questions:

RQ1: What AI technologies are currently applied for POPI prediction, monitoring, and prevention?

RQ2: What is the reported predictive or monitoring performance of these AI models and tools (e.g., AUC, sensitivity, specificity)?

RQ3: Which variables or risk factors are identified as key inputs for AI-based POPI prediction models?

RQ4: What clinical application outcomes have been reported for AI tools in POPI management, and how have they affected nursing practice and health system implementation?

### 2.2 Inclusion and exclusion criteria (PCC)

Eligibility criteria were defined using the PCC framework:

*Population*: Patients of any age or gender undergoing any type of surgical procedure. Studies focused primarily on non-surgical populations—for example, long-term bedridden ICU patients or community care recipients—were excluded.

*Concept*: The core concept was the use of AI technologies for POPI risk prediction, real-time monitoring, early detection, or decision support. AI technologies included but were not limited to machine learning (e.g., Random Forest, Support Vector Machine, XGBoost), deep learning (e.g., Convolutional Neural Networks, Recurrent Neural Networks), natural language processing, and algorithm-integrated hardware devices (e.g., intelligent pressure-sensing mats, thermographic systems). Eligible studies were required to apply a quantitative method for risk prediction, real-time monitoring, or decision support related to perioperative pressure injuries. Purely descriptive studies and those relying solely on univariate analysis without a predictive modeling component were excluded. No studies were excluded on the basis of algorithm choice alone (e.g., logistic regression versus machine learning); given the scoping nature of this review, we did not impose a strict boundary between traditional statistical methods and machine learning approaches. To ensure conceptual clarity, we established a tiered technology classification framework during the revision process to guide post-hoc synthesis:

- Tier 1 - Core Artificial Intelligence Algorithms: This category includes machine learning (e.g., Random Forest, XGBoost, Artificial Neural Network, Multilayer Perceptron, Alternating Decision Tree) and deep learning models, which represent the primary AI technologies of interest.
- Tier 2 - Conventional Statistical Benchmark Models: This category includes Logistic Regression-based predictive models. Although these are not AI algorithms per se, we retained them as benchmark comparators to provide a complete picture of the current quantitative predictive landscape, explicitly distinguishing them from Tier 1 throughout the synthesis.
- Tier 3 - Intelligent Sensing and Signal-Processing Systems: This category includes hardware-based monitoring systems (e.g., flexible pressure sensor arrays) combined with signal-processing techniques (e.g., Kalman filtering). These technologies are not AI algorithms in themselves, but serve as critical data acquisition and pre-processing layers for AI-driven real-time monitoring; they were included to capture the full technological pipeline relevant to AI-enabled perioperative care.

All included studies were classified into one of these three tiers during data extraction, and the synthesis was structured accordingly to prevent inappropriate conflation.

Context: Studies had to address the full perioperative workflow, encompassing the preoperative assessment area, operating room, post-anesthesia care unit (PACU), and early postoperative surgical ward. Studies set in home care, long-term care facilities, or non-acute rehabilitation centers were excluded.

Only peer-reviewed literature published in Chinese or English was included, encompassing original research articles, full-text conference papers (abstracts only were excluded), and dissertations. No lower date limit was applied, to ensure comprehensive coverage of this emerging field.

### 2.3 Search strategy

The search strategy followed the three-step process recommended by JBI (15). An initial limited search was first conducted in PubMed and Web of Science to identify relevant keywords and controlled vocabulary terms (e.g., MeSH, Emtree) across three core domains: (1) perioperative period (e.g., surgery, anesthesia, preoperative/intraoperative/postoperative); (2) pressure injury (e.g., pressure ulcer, decubitus ulcer); and (3) artificial intelligence and related technologies (e.g., machine learning, deep learning, sensors). The final search query was constructed by combining terms from these domains with the Boolean operator “AND.”

The following electronic databases were searched systematically from inception to December 11, 2025: PubMed, Embase, CINAHL (EBSCOhost), Cochrane Library, IEEE Xplore, ACM Digital Library, Web of Science Core Collection, and Scopus. Database-specific search strategies, adapted to each platform’s syntax, are provided in Supplementary material S1. Reference lists of included articles were also manually screened to supplement the electronic search.

### 2.4 Study selection and data extraction

All retrieved records were imported into EndNote X9 for duplicate removal. The remaining records were uploaded to the Rayyan online collaboration platform for screening. Screening proceeded in two stages: title and abstract review, followed by full-text assessment. Two reviewers (QZ and CY) independently conducted both stages. Disagreements were resolved through discussion and consensus; if consensus could not be reached, a third reviewer (JL) was consulted. The selection process and exclusion reasons at each stage are presented in a PRISMA-ScR flow diagram.

A standardized data extraction form was developed to collect information from the included studies. Extracted items included: ① basic study characteristics (author, year, country, study design); ② PCC elements (surgery type, perioperative stage, sample size); ③ AI technical specifications (algorithm or model type, key input variables, use of hardware sensors); ④ application objective (e.g., risk prediction, real-time monitoring); and ⑤ outcome measures (model performance metrics such as AUROC, sensitivity, and specificity; clinical outcomes such as changes in pressure injury incidence and user acceptance).

### 2.5 Considerations regarding quality appraisal

In line with JBI guidance for scoping reviews, the primary goal of this study was to map the breadth and characteristics of available evidence rather than to appraise its methodological quality or answer narrowly defined questions requiring high-level evidence synthesis (16). Accordingly, we did not conduct a formal quality assessment or risk-of-bias evaluation of the included studies—a decision consistent with standard scoping review practice. Our aim was to avoid excluding methodologically heterogeneous but content-relevant studies from an emerging field, which could obscure the exploratory mapping process. Instead, we addressed our research questions through comprehensive descriptive analysis of all eligible studies, thereby achieving the core objective of charting the evidence landscape and identifying research gaps. This pragmatic approach aligns with the evidence synthesis needs of public health decision-makers, who often require rapid, systematic mapping of nascent technologies to inform resource allocation and policy development, even when traditional high-level evidence remains limited.

### 2.6 Data synthesis and presentation

Given the anticipated heterogeneity in AI technologies, application contexts, and study designs, findings were synthesized using descriptive (narrative) analysis and evidence mapping.

- *Evidence mapping*: Studies were categorized according to their intervention points within the perioperative care pathway—retrospective/post-hoc risk modeling, intraoperative real-time monitoring, or postoperative early warning. Visual displays were created to illustrate the distribution of evidence across surgical specialties and technology types.
- *Narrative synthesis*: A summary description, structured around the four research questions, was developed to highlight AI model types, performance metrics, key predictive variables, and clinical implementation outcomes. Current trends, patterns, and areas of consensus were emphasized.
- *Gap identification*: Through systematic collation of the available evidence, gaps in the current knowledge base were delineated—including, for example, the absence of multicenter external validation, insufficient integration of intraoperative dynamic data, and limited evaluation of impact on clinical workflows. These gaps informed our recommendations for future research.

### 2.7 Data extraction content and form

The research team developed a standardized data extraction form using Excel. The form was pre-tested and refined on two relevant articles (not part of the final sample) before formal extraction. Given the dual clinical and technological nature of the topic, the following data were extracted

- *Basic characteristics*: Author(s), year, country, study design (e.g., retrospective cohort, randomized controlled trial, technology development).
- *Perioperative context*: Surgical type (e.g., cardiac, spinal), perioperative stage (preoperative, intraoperative, or postoperative), and sample size.
- *AI technical specifications*: (1) Algorithm or model type (e.g., Logistic Regression, Random Forest, Convolutional Neural Network); (2) input variables, including whether intraoperative dynamic variables (e.g., operative duration, hypotension, blood loss, body temperature) were incorporated; and (3) use of hardware devices such as sensors, cameras, or intelligent support surfaces.
- *Application objective*: Risk prediction, real-time monitoring, automated positioning adjustment, or image-based diagnosis.
- *Outcomes and performance*: Technical metrics (AUC, accuracy, sensitivity, specificity) and clinical metrics (changes in pressure injury incidence, alarm accuracy, user satisfaction).

## 3 Results

### 3.1 Search and screening results

A systematic search was conducted from database inception to December 11, 2025, in the following English-language bibliographic databases: PubMed, Embase, CINAHL (EBSCOhost), Cochrane Library, IEEE Xplore, ACM Digital Library, Web of Science Core Collection, and Scopus. Detailed search strings adapted to each database’s syntax are provided in Supplementary material S1.

The number of records retrieved per database was: ①PubMed: 137; ②Embase: 65; ③CINAHL: 183; ④Cochrane Library: 8; ⑤IEEE Xplore: 5; ⑥ACM Digital Library: 1; ⑦Web of Science: 233; ⑧Scopus: 219. After duplicate removal, 376 unique records remained. The complete screening process is reported in the PRISMA-ScR flow diagram (Figure 1).

**Figure 1:**
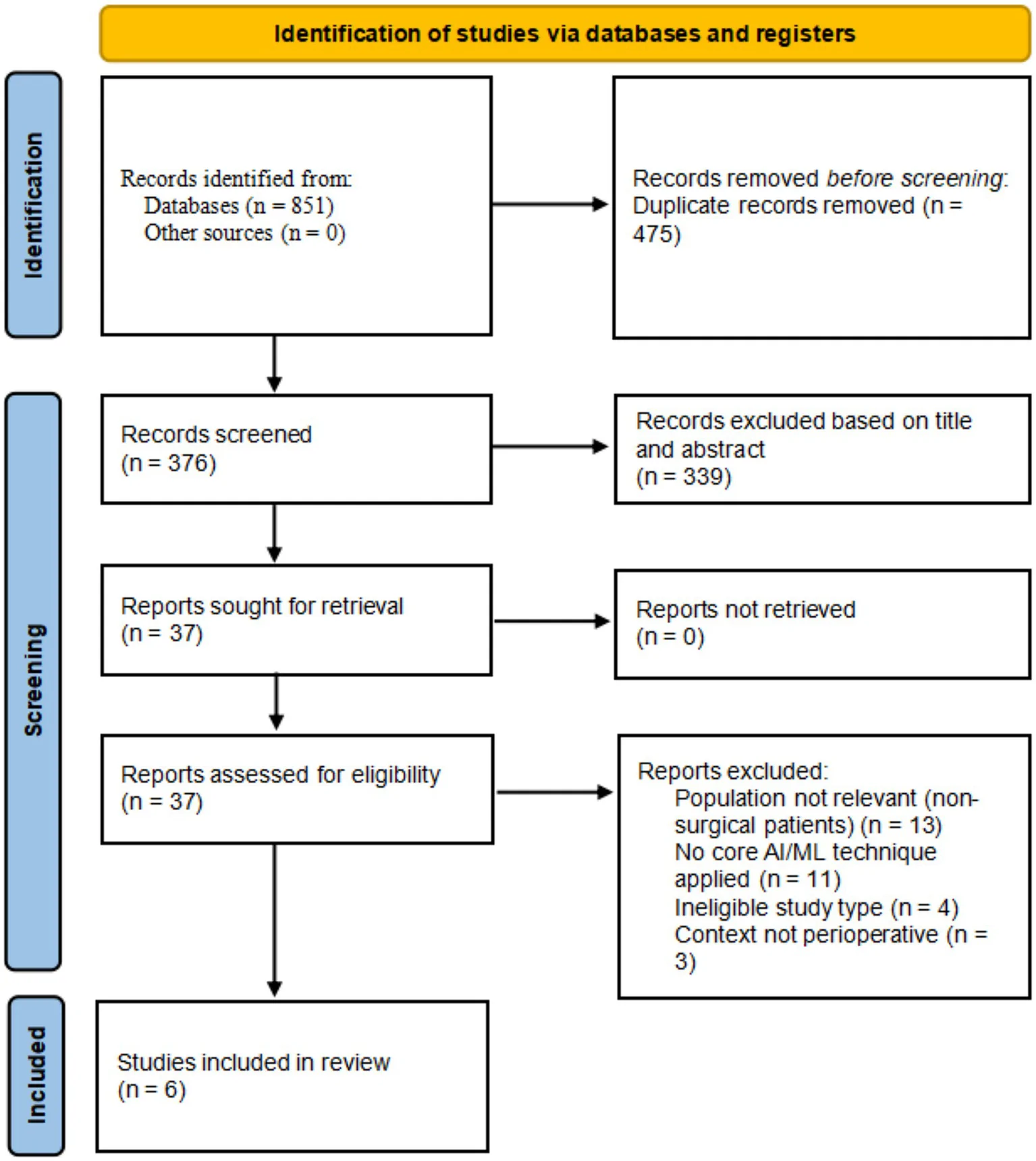
PRISMA-ScR Flow diagram of study selection.

### 3.2 Characteristics of included studies

The six included studies, published between 2018 and 2025, collectively reflect recent progress in applying AI to perioperative pressure injury management. Geographically, all originated from East Asia—five from China (Jiangsu, Beijing, and Taiwan) and one from Japan. This geographic concentration raises considerations regarding generalizability, which we address in the discussion. Five of the six studies were published within the last 5 years (2020–2025), suggesting growing research interest in this area.

All included studies used quantitative designs: retrospective cohorts (*n* = 2), prospective cohorts (*n* = 2), a cross-sectional study (*n* = 1), and one experimental study. The AI technologies employed were diverse, encompassing various machine learning models for POPI prediction (e.g., ANN, XGBoost, Random Forest, LR, MLP, DT), alongside one study using a flexible textile pressure sensing system with a Kalman filter for intraoperative monitoring.

Notably, although the five prediction studies were contextualized within the preoperative phase in their original reports, their predictor sets frequently included variables that only become available during or after surgery—such as operative duration, cardiopulmonary bypass time, intraoperative corticosteroid use, forced positioning, traction, and Trendelenburg positioning. We therefore reclassified these as perioperative or post-hoc risk stratification models based on the actual timing of predictor availability, rather than the surgical phase label. This reclassification is detailed in Table 1 and consistently applied throughout the synthesis.

**Table 1:** Basic characteristics of included studies (*n* = 6).

First author (Year)	Country	Study design	Study population	Sample size	Primary AI model/technology	Actual timing of predictor availability	Notes
Chen et al. (2018) (20)	China	Prospective cohort study	Cardiovascular surgical patients	149	Artificial Neural Network (ANN)	Post-hoc (using intraoperative/procedural variables)	149 cardiovascular surgical patients; secondary analysis of a prospective cohort
Shih et al. (2020) (19)	China	Experimental study	Cardiac surgery patients (intraoperative)	47	Flexible textile pressure sensor + Kalman filter	Intraoperative real-time	47 cardiac surgery patients (intraoperative monitoring)
Cai et al. (2020) (21)	China	Secondary analysis of a prospective cohort	Cardiovascular surgical patients	149	XGBoost	Post-hoc (using intraoperative/procedural variables)	149 cardiovascular surgical patients; secondary analysis of prospective cohort data
Zhao et al. (2024) (22)	China	Cross-sectional study	Adult orthopedic surgery patients	1,701	Random Forest	Post-hoc (using intraoperative/procedural variables)	1,701 orthopedic surgery patients
Shen et al. (2024) (18)	China	Prospective cohort study	Da Vinci robotic surgery patients	335	Logistic Regression	Post-hoc (using intraoperative/procedural variables)	335 Da Vinci robotic surgery patients
Li et al. (2025) (23)	China	Retrospective cohort study	Surgical patients	931	Multilayer Perceptron	Post-hoc (using intraoperative/procedural variables)	Initial matched cohort: 1,046 (523 pairs); 115 excluded; final analytical sample: 931

Sample sizes ranged from 47 to 8,286 patients, with data drawn from single-center or multicenter acute care hospitals. Details are presented in Table 1.

The study by Setoguchi et al. (17) was initially identified but excluded upon re-examination, as it analyzed a general hospital inpatient population (*n* = 12,008) rather than an exclusively surgical cohort. Although operation duration was included as a predictor, the study did not report an isolable perioperative subgroup and thus did not meet our prespecified surgical cohort criterion. All counts and analyses reflect the remaining six studies.

Funding sources were not consistently reported across the included studies; no study reported industry funding, and all declared no conflicts of interest relevant to the research.

### 3.3 Types and application modes of AI technologies

Based on the predefined tiered framework, the included studies fell into three methodological categories:

- Tier 1 - Machine learning-based predictive models (4 studies): These studies used retrospective data, incorporating both preoperative and intraoperative/procedural variables, to construct ML models for perioperative or post-hoc risk stratification. Algorithms included Artificial Neural Network (ANN), XGBoost, Random Forest, and Multilayer Perceptron (MLP).
- Tier 2 - Conventional regression-based predictive model (1 study): Shen et al. (18) used logistic regression to develop a nomogram for predicting POPI in patients undergoing Da Vinci robotic surgery, incorporating intraoperative variables including operative duration and Trendelenburg positioning. This study was retained as a benchmark comparator and is explicitly distinguished from Tier 1 models.
- Tier 3 - Sensor-based real-time monitoring with signal processing (1 study): Shih et al. (19) developed a flexible textile-based pressure sensor array with a Kalman filter, enabling continuous intraoperative pressure visualization. This system represents an enabling technology for AI-driven monitoring rather than an AI algorithm per se.
- Sensitivity Analysis by Technology Tier: To assess whether the inclusion of Tier 2 and Tier 3 affected our findings, we restricted the synthesis to Tier 1—core machine learning models only. Under this restricted definition, four studies remained [Chen et al. (20), Cai et al. (21), Zhao et al. (22), and Li et al. (23)]; the logistic regression study (18) and the pressure-sensing study (19) were excluded. The overall evidence pattern—predominance of retrospective prediction models, absence of external validation, and lack of postoperative early warning systems—remained unchanged. However, the intraoperative real-time monitoring evidence derived solely from the excluded Tier 3 study, indicating that this category represents a proof-of-concept breakthrough rather than a mature AI application. This sensitivity analysis confirms that our principal conclusions are robust to the inclusion of Tier 2 and Tier 3 technologies, but the intraoperative monitoring evidence remains contingent on a single, non-ML sensing study.

### 3.4 Key predictive variables

Across the included studies, the predictive variables used in AI models fell into several categories:

- *Patient demographics*: Age and Body Mass Index (BMI). Low BMI was frequently identified as an independent risk factor.
- *Surgery-related factors*: Operative duration, which multiple studies identified as a key risk factor.
- *Physiological and laboratory indicators*: Metrics reflecting nutritional status and perfusion, including serum albumin and hemoglobin levels.
- *Disease and treatment factors*: Comorbidities (e.g., diabetes) and intraoperative corticosteroid use.
- *Activity and mobility*: Transfer ability.

Most of these variables were extracted automatically from EHRs, illustrating the capacity of AI models to process multi-dimensional, high-frequency clinical data. Crucially, several of these predictors—including operative duration, cardiopulmonary bypass time, and intraoperative corticosteroid use—are only observable during or after the surgical procedure, reinforcing our reclassification of these models as post-hoc risk stratification rather than truly preoperative prediction.

### 3.5 Model performance

Performance metrics indicated that the included predictive models generally demonstrated moderate to promising internal discriminative ability, with variations across algorithms and study populations.

- *AUROC values*: For the best-performing model in each study with reported AUC, values ranged from 0.806 to 0.836 (Cai et al. (21): 0.806; Chen et al. (20): 0.815; Li et al. (23): 0.836). The Random Forest model in Zhao et al. (22) reported a prediction accuracy of 0.9733 in a single-center cross-sectional study of 1,701 orthopedic patients, rather than AUROC; this metric is not directly comparable to the AUC values reported in other studies. Direct cross-study comparisons should be interpreted with caution due to heterogeneity in populations, outcome definitions, sample sizes, and validation strategies.
- *Sensitivity and Specificity*: Varied across models. The XGBoost model by Cai et al. (21) achieved very high specificity (1.000) but relatively low sensitivity (0.0811), based on the original 2 × 2 table reported in the study (TP = 3, FN = 34, TN = 112, FP = 0); whereas the Multilayer Perceptron model by Li et al. (23) showed a different balance (sensitivity 0.688, specificity 0.851). Details are presented in Table 2.
- *Validation strategies*: None of the included prediction studies reported external validation. Internal validation strategies, when reported, included cross-validation or split-sample approaches, though specific methods were not consistently described. The performance estimates in Table 2 should therefore be interpreted as internal discriminative performance rather than generalizable accuracy.

**Table 2:** Performance metrics of predictive models in included studies (based on the best performing model reported).

First author (Year)	Best Model	AUROC	Sensitivity	Specificity	Positive predictive value	Negative predictive value	Notes
Chen et al. (2018) (20)	Artificial Neural Network	0.815	NR	NR	NR	NR	Internal validation via 70% training / 30% testing split; no external validation. Early application of ANN in cardiovascular surgery.
Shih et al. (2020) (19)	Flexible Sensing System	NR	NR	NR	NR	NR	Experimental study focused on real-time pressure monitoring and trend description; no classifier performance metrics were provided.
Cai et al. (2020) (21)	XGBoost	0.806	0.0811	1.000	1.000	0.7671	Based on the original 2 × 2 table (TP = 3, FN = 34, TN = 112, FP = 0); sensitivity = 3/37 = 0.0811; F1 score = 0.150. Internal validation method not specified; no external validation.
Zhao et al. (2024) (22)	Random Forest	N/A	NR	NR	NR	NR	Single-center cross-sectional study of 1,701 orthopedic patients. The reported value of 0.9733 is prediction accuracy, not AUROC; this metric should not be directly compared with AUC values from other studies. Internal validation method not specified; no external validation.
Shen et al. (2024) (18)	Logistic Regression	NR	NR	NR	NR	NR	The study focused on developing a nomogram prediction model; standard performance metrics were not provided.
Li et al. (2025) (23)	Multilayer Perceptron	0.836	0.688	0.851	NR	NR	Internal validation via 70% training / 30% testing split; no external validation. This study compared MLP, LR, and DT models simultaneously, with MLP performing the best.

NR, Not Reported. For Cai et al. (21), performance metrics were recalculated from the reported 2 × 2 contingency table (TP = 3, FN = 34, TN = 112, FP = 0) provided in the original study; F1 score = 0.150. For Zhao et al. (22), 0.9733 is prediction accuracy, not AUROC; direct comparison with AUC is not recommended.

### 3.6 Clinical application and implementation outcomes

Most studies focused on model development and validation and did not evaluate post-deployment effectiveness. One study, however, provided clinically relevant findings: Shih et al. (19) reported that among 47 cardiac surgery patients who eventually developed PIs, interface pressure at bony prominences—particularly the sacrococcygeal region—showed a marked upward trend after more than 2 hours of surgery. This observation points to a potential time window for intraoperative intervention.

### 3.7 Methodological characteristics of predictive models

To contextualize the reported AUC values, we summarized key methodological features of the five prediction studies. Sample sizes ranged from 47 to 1,701. All studies used internal validation strategies: three reported cross-validation, two used split-sample validation, and one did not specify the validation method. Calibration (e.g., Hosmer-Lemeshow test or calibration plots) was reported in only one study. None of the studies reported handling of missing data or addressed class imbalance explicitly. No study conducted external validation. This methodological heterogeneity underscores that the reported AUCs represent internal discriminative performance and should not be equated with generalizable clinical accuracy.

## 4 Discussion

This scoping review offers the first systematic evidence map, to our knowledge, that specifically applies a perioperative continuum framework and a population health and policy lens to AI-enabled POPI management, covering the full spectrum from prediction to monitoring. The discussion is organized around the perioperative pathway—preoperative, intraoperative, and postoperative—and addresses three interconnected aims: presenting the evidence map, synthesizing AI performance and key variables at each stage, and identifying research gaps.

Analysis of the six included studies shows that existing evidence is heavily concentrated on preoperative risk prediction, with early signs of intraoperative real-time monitoring, while postoperative early warning remains entirely uncharted. The discussion follows this workflow, concluding with an integrated translational pathway that maps the current knowledge landscape and points toward future research priorities. To maintain conceptual rigor, we structured our discussion according to the three-tier framework established in the Methods, explicitly distinguishing between ML-based predictions, conventional regression benchmarks, and sensor-based monitoring systems throughout the following sections.

### 4.1 Perioperative and post-hoc risk stratification: maturation of retrospective predictive models and the case for specialty-specific refinement

Most of the current evidence (five of six studies) centers on using historical EHR data, including intraoperative and procedural variables, to build ML models for POPI risk stratification. None of these models qualifies as a purely preoperative prediction tool using only data available before surgery; rather, they represent *post-hoc* risk stratification models that incorporate covariates observed during or after the procedure. This body of work demonstrates feasibility and preliminary internal discriminative accuracy for retrospective risk identification, but maturation will require external validation and impact assessment. Notably, none of the included prediction models underwent external validation or impact evaluation; their reported performance thus likely reflects optimistic estimates and should not be equated with generalizable clinical accuracy.

#### 4.1.1 Algorithm evolution and performance

The models have progressed from early ANN approaches to ensemble learning methods such as random Forest and XGBoost, and more recently to comparative multi-model studies. AUROC values for the machine learning prediction models that reported this metric ranged from 0.806 to 0.836 (Cai et al. (21): 0.806; Chen et al. (20): 0.815; Li et al. (23): 0.836). A random Forest model in a cross-sectional study of 1,701 orthopedic patients reported a prediction accuracy of 0.9733 (22), which is a different metric than AUROC and should not be directly compared with the AUC values reported in other studies.

Caution is warranted when comparing AUC values across studies, as the included prediction models differed substantially in population characteristics (general surgery vs. cardiovascular vs. orthopedic vs. robotic surgery), sample sizes (ranging from 47 to 1,701), outcome definitions, case-mix, and validation strategies. Critically, none reported external validation, and internal validation methods (e.g., cross-validation vs. hold-out) were not consistently described. The highest reported prediction accuracy of 0.9733 derives from a single-center cross-sectional study (22) and has not been tested in independent populations; therefore, it should not be interpreted as evidence of algorithmic superiority. Rather, the primary contribution of these prediction studies is to demonstrate proof-of-concept feasibility and identify specialty-specific risk factors warranting further prospective investigation. Future research must prioritize external validation, calibration assessment, and decision-curve analysis before claims of generalizable clinical utility can be made.

#### 4.1.2 Mining specialty-specific risks

A key contribution of this review is the finding that AI models can detect and weigh surgical-specific risk factors from high-dimensional EHR data. Some of these factors—operative duration and cardiopulmonary bypass time, for example—have been reported in the traditional POPI literature, but AI generates a quantified, multivariate risk profile that generic tools like the Braden scale cannot offer, given their static, non-surgical focus. Distinct specialty-specific patterns emerged: cardiopulmonary bypass time and intraoperative corticosteroid use were key in cardiovascular surgery (20); forced positioning and intraoperative traction figured prominently in orthopedic surgery (22); and prolonged operative time combined with the Trendelenburg position defined a unique risk profile in Da Vinci robotic surgery (18). These findings argue for developing specialty-specific AI prediction models. From a nursing and health systems perspective, perioperative assessments should move beyond generic scales and systematically incorporate procedure-specific risk variables to inform personalized prevention.

#### 4.1.3 Limitations and challenges

Although the included prediction models incorporate intraoperative covariates (e.g., operative duration, cardiopulmonary bypass time), they remain inherently static and retrospective—their inputs are fixed at model development and do not update dynamically as surgery proceeds. The true limitation is therefore not the absence of intraoperative data (which many models use), but two distinct gaps: (1) the absence of truly preoperatively deployable models using only pre-accessible variables, and (2) the absence of dynamically updatable real-time warning systems that integrate streaming intraoperative data to continuously modulate risk predictions.

Future work should pursue two parallel directions: (a) developing and externally validating prediction models restricted to truly pre-accessible variables (demographics, comorbidities, admission laboratory values) to enable preoperative risk stratification; and (b) constructing dynamic predictive frameworks that ingest multi-dimensional, real-time data streams—including interface pressure, hemodynamic parameters, and surgical milestones—to provide continuously updated risk alerts during surgery and the early postoperative period.

### 4.2 Intraoperative real-time monitoring: a paradigm shift from “invisible” to “Visualizable”

The intraoperative phase is the “black box” of POPI development—and a critical window for prevention. The study by Shih et al. represents a significant shift in this space (19). In contrast to the five post-hoc prediction studies that rely on retrospectively assembled covariates, this study exemplifies real-time intraoperative monitoring. This distinction underscores that intraoperative data are not absent from the literature—they appear as retrospective model inputs—but their use for dynamic, prospective risk warning remains almost entirely unexplored.

#### 4.2.1 Technological implementation and findings

Shih and colleagues developed a flexible textile-based pressure sensing system with a Kalman filter, achieving continuous, real-time monitoring of patient-table interface pressure in cardiac surgery. Among patients who eventually developed a PI, sacrococcygeal pressure rose markedly after more than 2 hours of surgery (19). This observation is hypothesis-generating and suggests a potential time window (approximately 2 hours) that warrants prospective testing in larger, multi-center cohorts; it does not, however, establish a validated intervention threshold or demonstrate that intervention at this time point reduces POPI incidence.

#### 4.2.2 Evidence scarcity and high potential

Only one real-time monitoring study has been published to date, highlighting a major research gap: how to combine predicted risk with real-time physiological and physical monitoring data to build a dynamic warning system. Future work should expand beyond pressure monitoring alone to incorporate multimodal data, including thermal imaging and wearable biosensors (24–26). For device developers and nursing researchers, the priority is to develop low-cost, sterile-compatible monitoring tools and translate their data into actionable intraoperative alerts.

### 4.3 Postoperative early warning: An unexplored frontier

None of the included studies examined AI-based early warning in the PACU or surgical ward during the postoperative period, a notable gap given that the early postoperative phase—when patients are recovering with limited mobility—represents a continuing risk period for POPI (27, 28).

Care during this phase currently depends largely on nursing judgment and scheduled assessments. The use of AI for dynamic risk prediction using postoperative vital signs, analgesic pump data, and early mobility measures is entirely unexplored (29, 30). Filling this gap would extend risk monitoring seamlessly from the operating room to the ward, closing the current discontinuity in intelligent prevention. Future nursing research should prioritize the development and validation of AI early-warning models based on continuous postoperative data streams.

### 4.4 Synthesizing the evidence map: clinical implications and translation pathway

Drawing on the preceding analysis, we developed a conceptual translational pathway for integrating AI into perioperative POPI management (Figure 2). This framework was informed by: (a) direct observations from the included studies (left panel), (b) established principles from the broader pressure injury prevention literature (external references), and (c) the authors’ reasoning based on identified evidence gaps (right panel). The framework is explicitly labeled as unvalidated and hypothesis-generating, and should not be interpreted as an evidence-based implementation guide.

**Figure 2:**
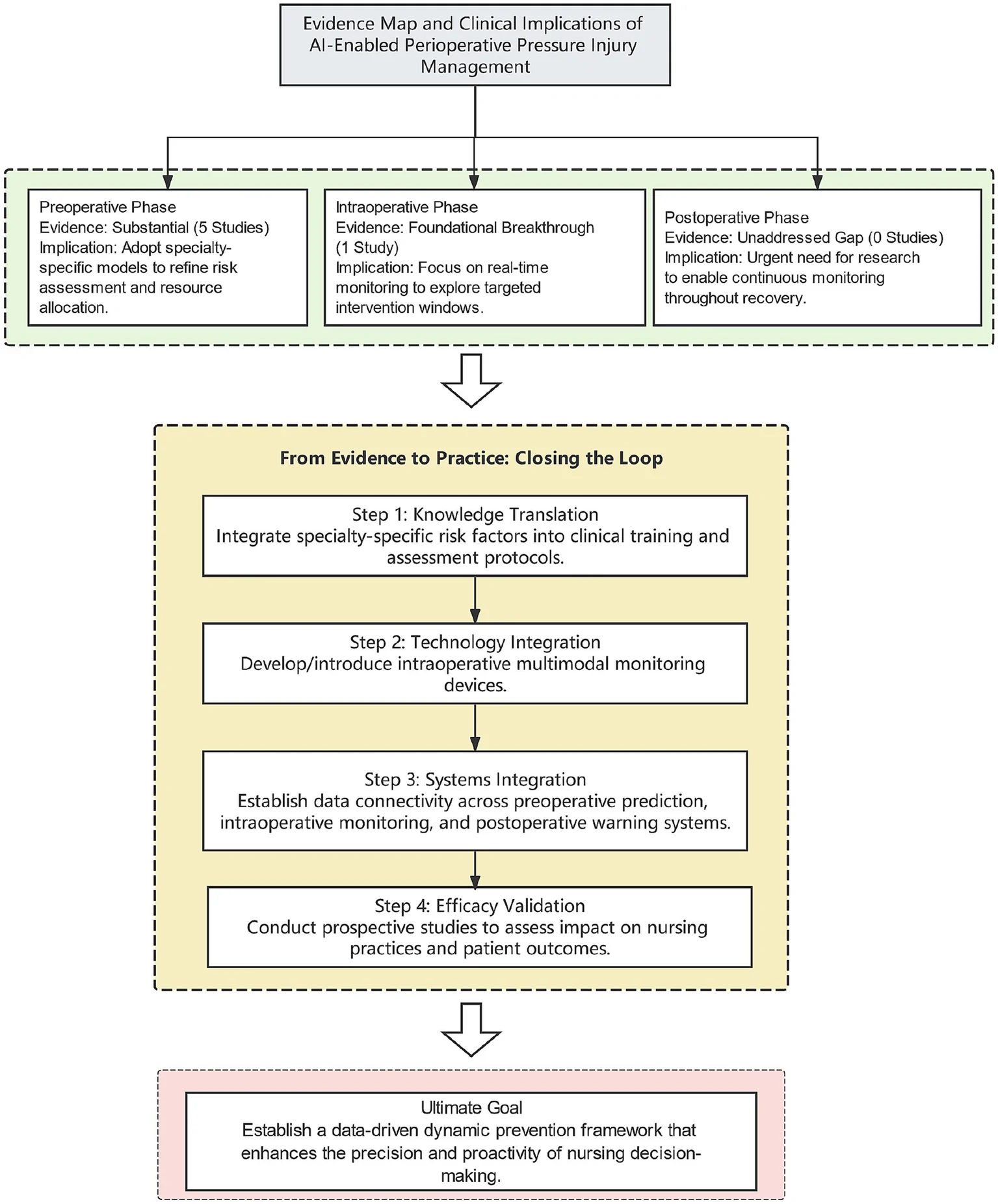
An unvalidated conceptual framework proposing a translational pathway for integrating AI and data-driven technologies into perioperative pressure injury management. The left panel synthesizes evidence-derived observations from the included studies. The right panel outlines hypothetical policy and implementation considerations extrapolated from the identified evidence gaps and the authors’ reasoning; these considerations have not been empirically tested and should be interpreted as a proposed research and policy agenda rather than evidence-based recommendations. The arrows illustrate the sequential translation from evidence mapping to clinical implementation and policy development.

#### 4.4.1 Summary of clinical implications

Clinical practice should prioritize the specialty-specific risk factors identified by AI research and incorporate them into perioperative nursing assessment protocols. The intraoperative pressure monitoring study suggests that preventive measures could be more precisely timed, and nursing protocols might incorporate reassessment checkpoints tied to surgical duration; however, this observation remains hypothesis-generating and requires prospective validation before protocol revision. Researchers should focus on the two underdeveloped areas—intraoperative monitoring and postoperative early warning—through both technological development and clinical studies (29–32).

#### 4.4.2 Future translation pathway

In the short term, well-performing specialty-specific prediction models should be validated and piloted in EHR systems to support preoperative risk assessment. In the mid-term, interdisciplinary collaboration should develop practical physiological and pressure-based monitoring technologies suitable for the operating room, with clinical pilot studies evaluating their effectiveness and impact on nursing workflows. In the long term, an intelligent data platform spanning the entire preoperative-intraoperative-postoperative continuum should enable seamless risk information transfer and linked early warnings, ultimately creating a closed-loop intelligent prevention system (33–35).

#### 4.4.3 Comparison with existing reviews and novelty of this work

To contextualize our contribution, we compared this scoping review with existing reviews on AI applications in pressure injury and perioperative care (Table 3).

**Table 3:** Comparison of the present scoping review with existing reviews on AI in pressure injury and perioperative care.

Review	Scope	Databases	Search date	Included studies	Technology definition	Quality assessment	Perioperative continuum focus	Population health/policy framing
Xu et al. (2025) (1)	Prediction models for intraoperative acquired pressure injury in adults	Not specified	2025	Not specified	Systematic review of prediction models	Critical appraisal	Partial (intraoperative only)	No
Toffaha et al. (2023) (9)	AI and decision support systems for hospital-acquired pressure injury prediction	Not specified	Not specified	Not specified	Broad (AI + machine learning + decision support systems)	Not specified	No (ICU/general ward)	No
Wei et al. (2025) (10)	AI in pressure injury diagnosis (image-based models, wound classification)	Not specified	2025	Not specified	AI/machine learning/deep learning	Critical appraisal	No	No
Bellini et al. (2024) (11)	AI/ML in operating room management (case duration prediction, PACU resource allocation, surgical scheduling)	Not specified	2024	Not specified	AI/machine learning	Not specified	Partial (OR only)	No
Arina et al. (2024) (13)	Machine learning for prediction of complications and prognostication in perioperative medicine	MEDLINE, Embase	2023	26 studies	Machine learning only (logistic regression excluded)	PROBAST	Partial (prediction only)	No
Mohammadi et al. (2024) (14)	Machine learning methods and hypotension prediction index for intraoperative hypotension prediction	Not specified	2024	26 studies	Machine learning only	PROBAST	Partial (intraoperative only)	No
Barghouthi et al. (2023) (36)	Machine learning for pressure injury prediction in hospitalized adults	6 databases (CINAHL, PubMed, Science Direct, IEEE, Cochrane, Google Scholar)	2017–2023	27 studies	Machine learning only (LR, DT, RF, SVM, NN, XGBoost)	JBI checklist	No (general/ICU populations)	No
Present review	AI and data-driven technologies in perioperative pressure injury management	8 databases (PubMed, Embase, CINAHL, Cochrane, IEEE Xplore, ACM Digital Library, Web of Science, Scopus)	December 2025	6 studies	Tiered (machine learning / logistic regression / sensor-based signal processing)	No (scoping review)	Full continuum	Yes

PI, pressure injury; OR, operating room; PACU, post-anesthesia care unit; PROBAST, Prediction model Risk Of Bias ASsessment Tool. For reviews where specific database names, search dates, or included study counts were not reported in the available abstract/summary, “Not specified” is indicated. Arina et al. (13) and Mohammadi et al. (14) used PROBAST for quality assessment; the present review, as a scoping review following JBI guidance, did not conduct formal quality appraisal.

As shown in Table 3, previous reviews addressed AI for pressure injury prediction in general hospital or ICU populations (9, 10), AI for operating room management (11), and machine learning for perioperative prediction more broadly (13, 14). None, however, focused specifically on the full perioperative continuum for pressure injury management, nor adopted a population health and digital public health policy framing. Our review is the first, to our knowledge, to systematically map evidence across all three perioperative phases using a perioperative continuum framework, classify technologies by functional roles (prediction vs. monitoring vs. early warning), and apply a digital public health policy lens to AI-enabled POPI management.

We acknowledge that the novelty of our contribution lies primarily in the perioperative continuum framing and the public health lens, rather than in the identification of individual AI prediction models, many of which have been discussed in previous reviews. Our evidence map reveals not the maturity of the field, but its fragmentation and the absence of integrated, dynamically updated systems across the perioperative care pathway.

### 4.5 Limitations and future directions

This review has several limitations. First, our search strategy was limited to English-language databases and did not include Chinese databases such as CNKI or Wanfang. Additionally, our search terms omitted emerging sensing-related keywords (e.g., pressure mapping, smart textile), which may have biased our findings toward prediction-model studies and away from sensor-based monitoring. The evidence map should therefore be viewed as illustrative rather than exhaustive.

Second, the small number of included studies (*n* = 6), all from East Asia, limits generalizability to broader populations. Our reclassification of prediction studies as perioperative/post-hoc risk stratification—based on the actual timing of predictor availability—was necessary due to the preoperative labeling in original reports, but this reclassification also highlights a broader limitation: the lack of standardized reporting guidelines for predictor timing in AI prediction studies. Additionally, one initially identified study (17) was excluded because its general hospital inpatient population did not meet our surgical cohort criterion; this exclusion may affect generalizability to surgical populations, though it enhances specificity to the perioperative context.

Third, we did not register *a priori* protocol, and we were unable to systematically describe internal validation methods due to inconsistent reporting across studies. While we have noted the absence of external validation, incomplete reporting of calibration, missing-data handling, and overfitting controls limits robust interpretation of the AUC values. We also did not conduct formal quality assessment using tools such as PROBAST, consistent with JBI guidance for scoping reviews (16), though this limits the ability to grade evidence strength.

Fourth, while we compared our work with existing reviews (Table 3), differences in inclusion criteria, search strategies, and technology definitions across reviews limit direct comparability; our contribution should therefore be viewed as complementary to, rather than replacing, prior syntheses.

Based on this evidence map, future research should: (1) conduct multi-center, prospective external validation of existing prediction models; (2) develop integrated multimodal sensor systems for intraoperative real-time monitoring and design dynamic warning algorithms linking real-time data with predictive models; (3) pioneer postoperative early warning systems using IoT and mobile health technologies; (4) strengthen interdisciplinary collaboration to embed AI tools into nursing workflows; and (5) improve search strategies in future reviews by including Chinese databases and broader AI- and sensing-related terms, with formal quality appraisal using tools such as PROBAST or TRIPOD-AI where applicable.

### 4.6 Future policy and health system considerations: a proposed agenda

The following considerations are proposed as a forward-looking agenda derived from identified evidence gaps, rather than conclusions directly supported by the six included studies. If future multi-center external validation confirms the generalizability of current prediction models, investments in EHR infrastructure, data standardization, and regulatory frameworks for algorithm auditing will become necessary. User-centered implementation strategies—such as embedding AI-generated risk scores into existing clinical workflows—should be prioritized to minimize documentation burden. Policymakers should also consider governance mechanisms for algorithm transparency, regular calibration updates, and clear protocols for human override of AI recommendations. These considerations, however, are contingent upon prospective validation and cost-effectiveness analyses, none of which have been reported in the current literature.

## 5 Conclusion

This scoping review demonstrates that AI and data-driven technologies in perioperative pressure injury management are at an early, transitional stage. Current evidence is geographically concentrated in East Asia—highlighting an unassessed risk regarding generalizability and algorithmic fairness—and predominantly retrospective, focusing on post-hoc risk stratification using intraoperative covariates. While prediction models show promising internal discriminative performance (AUC 0.806–0.836 for machine learning models, with one study reporting a prediction accuracy of 0.9733), none has undergone external validation, and clinical effectiveness remains unproven. Intraoperative real-time monitoring has emerged as a proof-of-concept breakthrough, yet evidence is confined to a single small study, and postoperative early warning systems are entirely absent.

The major contribution of this review is not to establish effectiveness, but to systematically expose the sparse, heterogeneous, and largely internally-validated nature of the evidence base, and to chart a prioritized research agenda—particularly for multi-center external validation, dynamic intraoperative monitoring, postoperative early warning, and assessment of algorithmic fairness across diverse populations. For clinicians, the priority is to recognize AI-identified specialty-specific risk factors as hypothesis-generating inputs for assessment protocols while awaiting prospective validation. For health policymakers, investments should target implementation science and cost-effectiveness evaluations rather than immediate large-scale deployment. Advancing this field ultimately requires coordinated interdisciplinary efforts to transform reactive wound care into a precise, proactive, and equitable model of perioperative population health protection—but this vision remains contingent on substantially stronger empirical evidence than currently available.

## Data Availability

The original contributions presented in the study are included in the article/Supplementary material, further inquiries can be directed to the corresponding author.
