# Supplementary material for: Artificial intelligence in perioperative pressure injury management: a scoping review of evidence, gaps, and implications for population health and digital public health policy
Source: Front Public Health. 2026 Oct 1;14:1952864. doi: 10.3389/fpubh.2026.1952864 (PMC13668841; doi:10.3389/fpubh.2026.1952864)
Supplement: Supplementary file 1 [file Table_1.DOCX]

**Supplementary Material 1.** Search strategy in selected databases

| Database  (Host) | Search String | Results |
| --- | --- | --- |
| PubMed | ((("Perioperative Care"[Mesh] OR "Surgical Procedures, Operative"[Mesh] OR "Intraoperative Period"[Mesh] OR "Anesthesia"[Mesh] OR perioperative*[Title/Abstract] OR intraoperative*[Title/Abstract] OR preoperative*[Title/Abstract] OR postoperative*[Title/Abstract] OR surgery[Title/Abstract] OR surgical[Title/Abstract] OR surgeries[Title/Abstract] OR anesthesia*[Title/Abstract] OR "operating room"[Title/Abstract]))  AND  (("Pressure Ulcer"[Mesh] OR "pressure injury"[Title/Abstract] OR "pressure injuries"[Title/Abstract] OR "pressure ulcer*"[Title/Abstract] OR "decubitus ulcer*"[Title/Abstract] OR bedsore*[Title/Abstract] OR "pressure sore*"[Title/Abstract] OR "skin breakdown"[Title/Abstract]))  AND  (("Artificial Intelligence"[Mesh] OR "Machine Learning"[Mesh] OR "Deep Learning"[Mesh] OR "Neural Networks, Computer"[Mesh] OR "artificial intelligence"[Title/Abstract] OR "machine learning"[Title/Abstract] OR "deep learning"[Title/Abstract] OR "neural network*"[Title/Abstract] OR "computer vision"[Title/Abstract] OR algorithm*[Title/Abstract] OR "predictive model*"[Title/Abstract] OR "random forest"[Title/Abstract] OR "support vector machine"[Title/Abstract] OR "decision support system*"[Title/Abstract] OR sensor*[Title/Abstract] OR wearable*[Title/Abstract]))) | 137 |
| Embase | ('perioperative period'/exp OR 'surgery'/exp OR perioperative:ti,ab OR surgical:ti,ab OR intraoperative:ti,ab OR postoperative:ti,ab)  AND  ('decubitus'/exp OR 'pressure injury':ti,ab OR 'pressure ulcer':ti,ab OR 'bedsore':ti,ab)  AND  ('artificial intelligence'/exp OR 'machine learning'/exp OR 'deep learning'/exp OR 'neural network':ti,ab OR algorithm*:ti,ab OR 'predictive modeling':ti,ab) | 65 |
| CINAHL (EBSCOhost) | S1 (MH "Perioperative Care+") OR (MH "Surgical Procedures, Operative+") OR (MH "Anesthesia+") OR TI (perioperative* OR intraoperative* OR preoperative* OR postoperative* OR surg* OR anesthesia*) OR AB (perioperative* OR intraoperative* OR preoperative* OR postoperative* OR surg* OR anesthesia*)  S2 (MH "Pressure Ulcer+") OR TI ("pressure injur*" OR "pressure ulcer*" OR "pressure sore*" OR bedsore* OR decubitus) OR AB ("pressure injur*" OR "pressure ulcer*" OR "pressure sore*" OR bedsore* OR decubitus)  S3 (MH "Artificial Intelligence+") OR (MH "Machine Learning+") OR (MH "Wearable Technology+") OR TI ("artificial intelligence" OR "machine learning" OR "deep learning" OR "neural network*" OR algorithm* OR "predictive model*" OR sensor* OR "smart device*") OR AB ("artificial intelligence" OR "machine learning" OR "deep learning" OR "neural network*" OR algorithm* OR "predictive model*" OR sensor* OR "smart device*")  S4 S1 AND S2 AND S3 | 183 |
| Cochrane Library | (perioperative OR intraoperative OR preoperative OR postoperative OR surgery OR surgical OR anesthesia) AND ("pressure injury" OR "pressure ulcer" OR bedsore* OR decubitus) AND ("artificial intelligence" OR "machine learning" OR "deep learning" OR "neural network*" OR algorithm*) | 8 |
| IEEE Xplore | ("pressure injury" OR "pressure ulcer" OR bedsore) AND (perioperative OR surgery) AND ("artificial intelligence" OR "machine learning" OR algorithm OR sensor) | 5 |
| ACM Digital Library | (Title:("pressure injury" OR "pressure ulcer" OR bedsore) OR Abstract:("pressure injury" OR "pressure ulcer" OR bedsore))  AND  (Abstract:(perioperative OR surgery OR intraoperative OR preoperative OR postoperative))  AND  (Abstract:("artificial intelligence" OR "machine learning" OR "deep learning" OR algorithm OR sensor)) | 1 |
| Web of Science | TS=(perioperative* OR intraoperative* OR preoperative* OR postoperative* OR surgery OR surgical OR anesthesia*)  AND  TS=("pressure injury" OR "pressure injuries" OR "pressure ulcer" OR "pressure ulcers" OR "pressure sore*" OR bedsore* OR decubitus)  AND  TS=("artificial intelligence" OR "machine learning" OR "deep learning" OR "neural network*" OR algorithm* OR "predictive model*" OR sensor* OR "computer vision" OR "smart mattress") | 233 |
| Scopus | TITLE-ABS-KEY(perioperative* OR intraoperative* OR preoperative* OR postoperative* OR surgery OR anesthesia*)  AND  TITLE-ABS-KEY("pressure injury" OR "pressure ulcer" OR bedsore* OR decubitus)  AND  TITLE-ABS-KEY("artificial intelligence" OR "machine learning" OR "deep learning" OR "neural network*" OR algorithm* OR "predictive model*" OR sensor* OR wearable*) | 219 |
